# Methods to assess binocular rivalry with periodic stimuli

**DOI:** 10.1186/s13408-020-00087-8

**Published:** 2020-06-15

**Authors:** Farzaneh Darki, James Rankin

**Affiliations:** Department of Mathematics, College of Engineering, Mathematics & Physical Sciences, University of Exeter, Exeter, UK

**Keywords:** Bifurcation analysis, Periodic forcing, Rivalry model, Traditional rivalry, Flicker and switch rivalry

## Abstract

Binocular rivalry occurs when the two eyes are presented with incompatible stimuli and perception alternates between these two stimuli. This phenomenon has been investigated in two types of experiments: (1) Traditional experiments where the stimulus is fixed, (2) eye-swap experiments in which the stimulus periodically swaps between eyes many times per second (Logothetis et al. in Nature 380(6575):621–624, 1996). In spite of the rapid swapping between eyes, perception can be stable for many seconds with specific stimulus parameter configurations. Wilson introduced a two-stage, hierarchical model to explain both types of experiments (Wilson in Proc. Natl. Acad. Sci. 100(24):14499–14503, 2003). Wilson’s model and other rivalry models have been only studied with bifurcation analysis for fixed inputs and different types of dynamical behavior that can occur with periodically forcing inputs have not been investigated. Here we report (1) a more complete description of the complex dynamics in the unforced Wilson model, (2) a bifurcation analysis with periodic forcing. Previously, bifurcation analysis of the Wilson model with fixed inputs has revealed three main types of dynamical behaviors: Winner-takes-all (WTA), Rivalry oscillations (RIV), Simultaneous activity (SIM). Our results have revealed richer dynamics including mixed-mode oscillations (MMOs) and a period-doubling cascade, which corresponds to low-amplitude WTA (LAWTA) oscillations. On the other hand, studying rivalry models with numerical continuation shows that periodic forcing with high frequency (e.g. 18 Hz, known as flicker) modulates the three main types of behaviors that occur with fixed inputs with forcing frequency (WTA-Mod, RIV-Mod, SIM-Mod). However, dynamical behavior will be different with low frequency periodic forcing (around 1.5 Hz, so-called swap). In addition to WTA-Mod and SIM-Mod, cycle skipping, multi-cycle skipping and chaotic dynamics are found. This research provides a framework for either assessing binocular rivalry models to check consistency with empirical results, or for better understanding neural dynamics and mechanisms necessary to implement a minimal binocular rivalry model.

## Introduction

In bistable perception, our perceptual experience evolves dynamically with fixed sensory inputs, thus providing a window into the intrinsic neural computations underlying the dynamics of sensory processing. Binocular rivalry is one example from a broad range of bistable or multistable perceptually ambiguous stimuli. It occurs when the two eyes are presented with incompatible monocular patterns (exclusively to one eye). Even though the patterns are fixed, perceptual awareness alternates every 1–5 s between two perceptual interpretations consistent with only one monocular pattern [3]. In other words, one of the monocular patterns is suppressed totally and the other one is dominant (thus winning the rivalry). This phenomenon has been investigated in a wide range of experimental approaches: human behavioral studies [1, 4], primate physiological experiments [5, 6], fMRI (functional magnetic resonance imaging) studies [7, 8], and EEG (electroencephalogram) recordings in humans [9]. Despite a long tradition of binocular rivalry research there are still questions about the neuro-computational basis and the underlying mechanisms of this phenomenon. In addition to experimental investigations, powerful tools such as mathematical modeling and bifurcation analysis have been utilized to explain this phenomenon [10–12], but many interesting questions remain.

There are contradictory ideas about whether rivalry takes place between neural populations associated with each eye or associated with stimulus features [1, 13]. The complex hierarchical architecture of visual cortical areas makes it difficult to associate object perception to a particular locus in cortex. The locus of visual perception and the level at which binocular rivalry is resolved are unknown. Previously, primary visual cortex (V1) had been considered as the locus of rivalry alternations and the phenomenon known as interocular eye rivalry [14–16]. According to this traditional interpretation, perceptual rivalry occurs as a result of competition between monocular driven neurons in primary visual cortex (V1) (eye-based rivalry). On the contrary, there are some studies which show correlation between perception during rivalry and activity in feature-selective higher cortical areas whose inputs are pooled from both eyes (supporting stimulus rivalry) [5, 6]. In order to resolve this dispute, Logothetis et al. introduced a new stimulus paradigm, so-called Flicker and Swap (F&S) in which monocular stimuli were swapped between two eyes at 1.5 Hz (eye-swapping). The stimuli were flickered on and off at 18 Hz in order to reduce the subject’s awareness of the swap times [1]. The notion of eye rivalry predicts that a subject’s perception must follow the stimulus as it switches between the eyes. However, F&S experiments, similar to traditional experiments, showed that each period of perceptual dominance lasts on average around 2 seconds and spans over six to seven stimulus swaps [1]. It can be concluded from the F&S experiment that rivalry cannot be the result of suppressing one eye completely (and dominance of the other eye). These results suggest that the stimulus rivalry hypothesis is a more accurate interpretation than eye rivalry.

For decades, the common empirical paradigm for studying binocular rivalry has been through stimulation of each eye with fixed stimuli (always on) [17, 18]. Several computational models of binocular rivalry have been proposed that were able to capture temporal characteristics of traditional rivalry (eye-based) with a specific set of model parameters [10–12, 19–23]. The minimal properties required to implement a model that is compatible with the existing rivalry evidence is reciprocal inhibition between two monocular neural populations and a slow process (spike-frequency adaptation or synaptic depression) together with nonlinearity of the spike rate gain function [21]. Although these models posed different features in the implementation of reciprocal inhibition, slow processes and gain function, all of them show common qualitative characteristics for neural competition [11].

**Figure 1:**
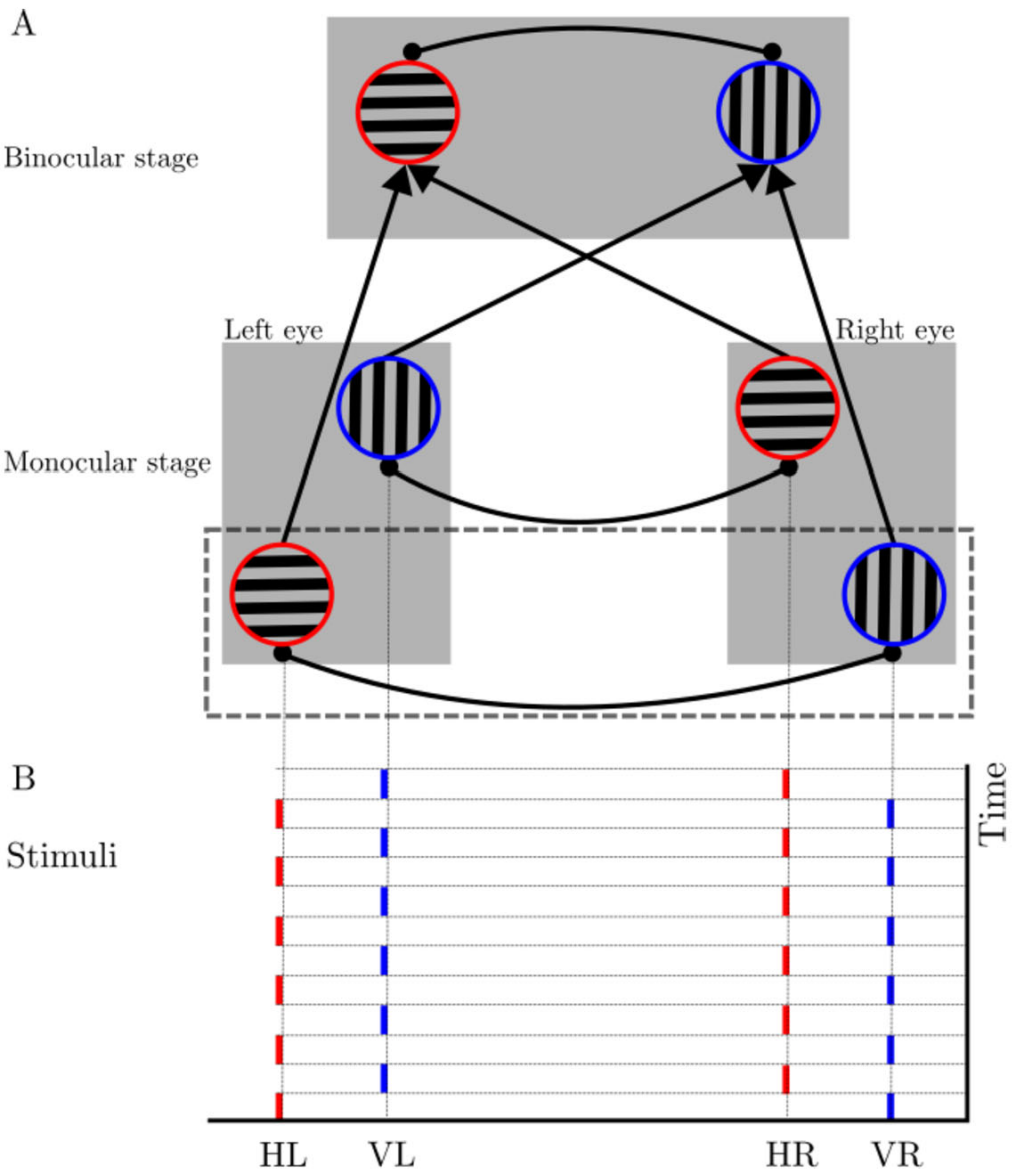
Two stage Wilson neural network model. (**A**) The first stage comprises monocular left and right neurons selective to orthogonal gratings. Reciprocal inhibition between different eyes and grating orientations are represented by heavy lines with filled circles at the ends. The second stage represents binocular neurons in higher cortical layers that receive the summation of left and right monocular neurons with the same grating orientation [2]. The isolated units at the first stage that we analyse are marked with the dashed box. (**B**) Horizontal (H) and vertical (V) stimuli swapped between the left (L) and the right (R) eyes (no flicker). At a specific time, one eye receives horizontal stimuli and the other receives vertical stimuli, each stimulates their own corresponding population at the first stage

Wilson sought to explain the stimulus rivalry with a two-stage model [2]. The first stage represents monocular neurons in primary visual cortex, and the second stage represents binocular neurons in later (higher) stages of processing (Fig. 1(A)). The Wilson model can account for maintained perceptual dominance across eye swaps with F&S stimuli [2]. However, this model predicts that monocular neural activity is not modulated during stimulus rivalry, which is contradicted by experimental evidence [24]. Whilst a later paper from Wilson [21] suggested these could be accounted for by descending connections to the monocular layer, Brascamp et al. claimed that it is possible to have stimulus rivalry without binocular contributions [24]. The Li et al. model [25] similar to the Wilson model [2] included a second stage to explain stimulus rivalry; however, unlike the Wilson model this second layer is responsible for attentional modulation rather than another layer of rivalry. Attentional modulation provides feedback to monocular layers, and thus stimulus rivalry can be seen in the first layer. Li et al. [25] also note that stimulus rivalry can occur without flicker in experiments if there are blank intervals before swaps times [26, 27], which was not captured by the Wilson model.

Traditional rivalry with a fixed stimulus in each eye was studied with the Wilson model [2]. Earlier work presented a bifurcation analysis of the Wilson model with fixed inputs and identified parameter regions with different dynamical behavior [11]. However, we found that a richer bifurcation structure exists at the crucial transition from winner-take-all to rivalry. Thus, a bifurcation analysis for fixed inputs that investigates all bifurcations occurring between different dynamical states is required. Furthermore, Wilson’s model and other rivalry models have been only studied using bifurcation analysis for fixed inputs, whilst the different dynamical behavior that can occur with periodically forced inputs have only been investigated at isolated parameter values with direct simulations (time histories) [2], although see [28]. In general, sensitivity to model parameters with periodic inputs has not been studied so far. To fully explain F&S experiments, we need to understand the bifurcations that distinguish different states such as perception following the stimulus swaps and perception being stable for many seconds. Thus, a complete bifurcation analysis of the model with fixed and periodic inputs with careful attention to complex dynamics phenomena is required.

We aim to properly explain and understand the dynamics of binocular rivalry and how they change with different kinds of inputs (traditional, flickering, swapping, and F&S stimuli). Here we focus on the Wilson model and we were successful in building a framework to study rivalry models with, not only fixed input, but also with periodic forcing. For a thorough dynamical analysis, we analyse the Wilson model with periodic input using numerical continuation tools. This allows us to investigate different dynamical regimes and boundaries between them (bifurcations), as computed for multiple parameters. The approach presented here is applicable to a range of models for bistable perception with periodically varying stimuli.

## Methods

Here we focus on the two-stage, hierarchical Wilson model which can explain both traditional and F&S experiments [2]. The first stage represents monocular neurons in primary visual cortex, and the second stage represents binocular neurons in higher cortical areas (Fig. 1(A)). These neurons self-adapt, as modelled by spike-frequency adaptation, and there is mutual inhibition between monocular neurons representing different eyes and grating orientations. We split the first level into two isolated subunits:

1. competition between neurons representing the horizontal grating in the left eye (HL) and the vertical grating in the right eye (VR).
2. competition between neurons representing the vertical grating in the left eye (VL) and the horizontal grating in the right eye (HR).

Spike-rate equations for the first isolated subunit in the first stage of the Wilson model [2] are given by

1 $$ \begin{aligned} & \tau {\dot{E}}_{\mathrm{HL}} = - {E_{\mathrm{HL}}} + \frac{100(J_{\mathrm{HL}}(t)-gI_{\mathrm{VR}})_{+}^{2}}{(10+{H_{\mathrm{HL}}})^{2}+(J_{\mathrm{HL}}(t)-gI_{\mathrm{VR}})_{+}^{2}}, \\ & {\tau _{H}} {{\dot{H}}_{\mathrm{HL}}} = - {H_{\mathrm{HL}}} + {h} {E_{\mathrm{HL}}}, \\ & {\tau _{I}} {{\dot{I}}_{\mathrm{HL}}} = - {I_{\mathrm{HL}}} + {E_{\mathrm{HL}}}, \\ & {{\tau } {\dot{E}}_{\mathrm{VR}}} = - {E_{\mathrm{VR}}} + \frac{100(J_{\mathrm{VR}}(t)-gI_{\mathrm{HL}})_{+}^{2}}{(10+{H_{\mathrm{VR}}})^{2}+(J_{\mathrm{VR}}(t)-gI_{\mathrm{HL}})_{+}^{2}}, \\ & {\tau _{H}} {{\dot{H}}_{\mathrm{VR}}} = - {H_{\mathrm{VR}}} + {h} {E_{\mathrm{VR}}}, \\ & {\tau _{I}} {{\dot{I}}_{\mathrm{VR}}} = - {I_{\mathrm{VR}}} + {E_{\mathrm{VR}}}. \end{aligned} $$

Here $J_{\mathrm{HL}}$ and $J_{\mathrm{VR}}$ are inputs to populations representing a horizontal grating in the left eye and a vertical grating in the right eye, respectively. $E_{i}$ is the firing rate of the excitatory population *i* ($i={\mathrm{HL}},{\mathrm{VR}} $), $H_{i}$ is the adaptation variable, and $I_{i}$ is the inhibitory firing rate. The asymptotic firing rate, the second term on the right-hand side of the first and fourth expressions in (1), is determined by a Naka–Rushton function for positive values of its argument $(J-gI)_{+}$, where $(J-gI)_{+}=J-gI$ if $J\geq gI$ and $(J-gI)_{+}=0$ if $J < gI$ (Naka and Rushton 1966). The following values of the parameters are used: $\tau = 20\mbox{ ms}$, $\tau _{H}= 900\mbox{ ms}$, $\tau _{I}= 11\mbox{ ms}$, as in the original paper [2]. The values of inhibition strength *g* and adaptation strength *h* are varied as part of the bifurcation analysis.

Here we only consider one of the isolated subunits in the first stage (marked by a dashed box in Fig. 1(A)). By this simplification, we will only have two populations of neurons which correspond to the monocular neurons sensitive to horizontal stimuli in the left eye (HL) and vertical stimuli in the right eye (VR). For the traditional experiment, we will not lose generality since each eye only receives horizontal or vertical stimuli and one of the subunits always has no inputs. For the periodically forced cases, both subunits have their own inputs (Fig. 1(B)). However, because of symmetry and the feed-forward nature of the network we can carry out our analysis without the other subunit at monocular layer. We only investigate one of the subunits, which is further justified and considered along with the implications of our results for the full model in the discussion.

**Figure 2:**
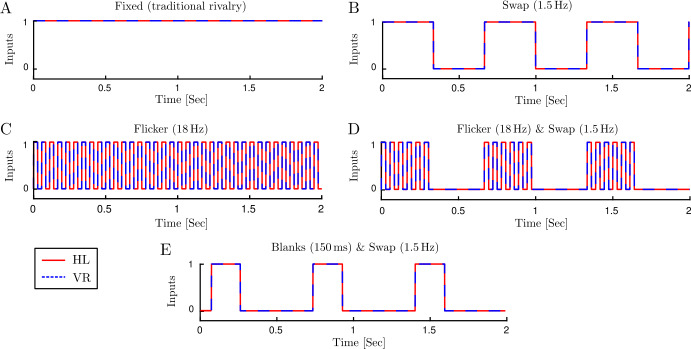
Stimuli for different input cases. Stimuli for monocular populations selective to the horizontal grating in the left eye (HL) and vertical gratings in the right eye (VR) are shown with red solid and blue dashed lines, respectively. (**A**) Fixed inputs for traditional rivalry. (**B**) Periodic 1.5 Hz square waves for swap only. (**C**) Periodic 18 Hz square waves for flicker only. (**D**) Periodic 1.5 Hz square waves modulated with 18 Hz on/off switches for F&S. (**E**) Periodic 1.5 Hz square waves with blank intervals (150 ms duration) inserted before swaps for the B&S experiment

Stimuli for different cases: traditional rivalry, swap only, flicker only, F&S, and B&S are shown in Fig. 2. In order to produce periodic forcing stimuli, we add two ODEs to the main equations in (1), which describe a nonlinear oscillator,

2 $$ \begin{aligned} & \dot{x}_{s} = x_{s} + (2 \pi f_{s} ) y_{s}-x_{s} \bigl(x_{s}^{2}+y_{s}^{2}\bigr), \\ & \dot{y}_{s} = - (2 \pi f_{s} ) x_{s}+y_{s}-y_{s} \bigl(x_{s}^{2}+y_{s}^{2}\bigr), \end{aligned} $$

with solutions: $x_{s}(t) = \sin (2 \pi f_{s} t)$, $y_{s}(t) = \cos (2 \pi f_{s} t) $, where $f_{s}$ is the frequency of oscillations. In order to have a smooth square form wave rather than sinusoidal, we use a steep sigmoid,

3 $$ x(t) = \frac{1}{{1+e^{ -k x_{s}(t)}}}, $$

with $k=10 $. Thus our desirable periodic forcing for swap only ($f_{s}=1.5 \mbox{ Hz} $) and flicker only ($f_{s}=18\mbox{ Hz} $) cases can be applied by replacing $J_{\mathrm{HL}}(t)=J_{\mathrm{VR}}(t)=x(t) $ in (1). For F&S stimuli, we have two forcing terms with different frequencies. Considering that the flickering frequency is an integer multiple of swap frequency ($f_{f}=12 f_{s} $), we first build the lower frequency forcing ($x_{s} $) using Eqs. (2), and the higher frequency forcing ($x_{f} $) can be computed by

4 $$ x_{f}(t)=\operatorname{Re}\bigl\{ \bigl[x_{s}(t)+iy_{s}(t) \bigr]^{12}\bigr\} . $$

Then the F&S stimuli can be produced from its components:

5 $$ x_{fs}(t)=x_{f}(t)x_{s}(t). $$

The nonlinear gain function that appears in the right-hand side of the original Wilson model is a discontinuous function in its first derivative due to the rectification operation $(J-gI)_{+} $. Numerical continuation routines require smooth systems of equations. In order to solve this problem we have used a steep sigmoid function to smooth out the transition at zero. So instead of the $(J-gI)_{+} $ terms in (1), we substitute $R(J-gI) $ as:

6 $$ R(x) = \frac{x}{{1+e^{ -k (x-\theta )}}}. $$

Where $k=30 $ defines the slope and $\theta =0.05 $ defines the threshold. This becomes particularly important when we want to follow the locus of torus bifurcations (T) in the parameter plane. Further difficulties in following the locus of torus bifurcations were resolved by introducing a small parameter ($\epsilon =0.001 $) to break the $\mathrm{HL} \leftrightarrow \mathrm{VR}$ symmetry in the first ODE in (1),

7 $$ \tau {\dot{E}}_{\mathrm{HL}} = - {E_{\mathrm{HL}}} + \frac{100(J_{\mathrm{HL}}(t)-gI_{\mathrm{VR}})_{+}^{2}}{(10+{H_{\mathrm{HL}}+\epsilon })^{2}+(J_{\mathrm{HL}}(t)-gI_{\mathrm{VR}})_{+}^{2}}. $$

This removed a potential degeneracy in the torus bifurcation defining system that allowed for the computations to be completed. The numerical integration of the initial value problems were carried out in MATLAB using a fourth-order Runge–Kutta method with time step 0.1 ms. Numerical continuation was performed with the package AUTO07p, by and large using relatively standard constants, but notably increasing the mesh size (NTST) for the computation of periodic orbits in the forced cases and when the period became large [29]. Source code for the model is available in the GitHub repository farzaneh-darki/Darki2020_methods: https://github.com/farzaneh-darki/Darki2020_methods.

## Results

### Bifurcation analysis of traditional rivalry with fixed inputs

**Figure 3:**
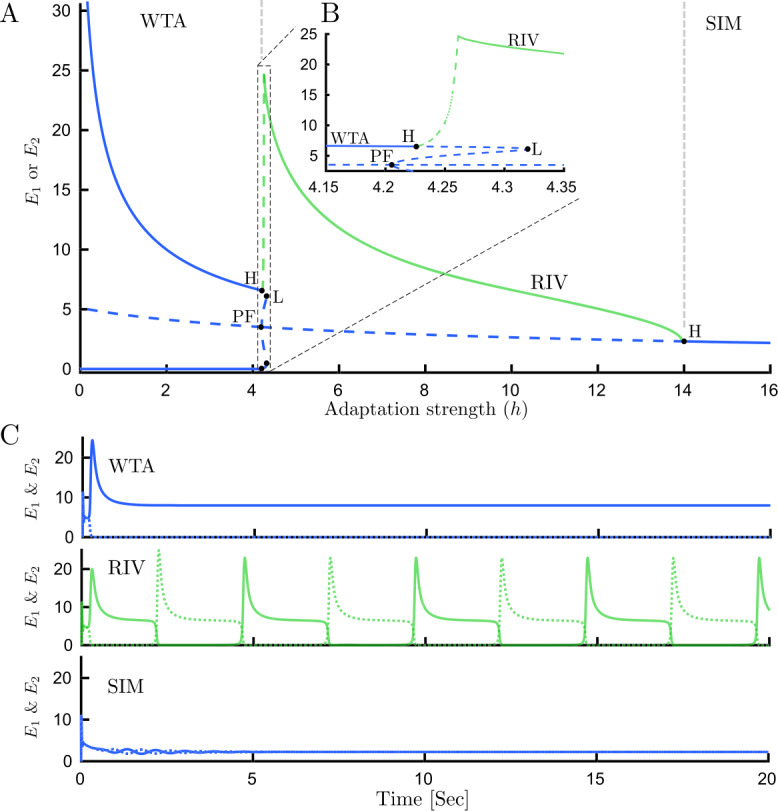
Bifurcation analysis and time histories for traditional rivalry. (**A**) Bifurcation diagram for the Wilson model (1) with fixed inputs varying adaptation strength *h*. Three main types of dynamical behaviors are presented: Winner-takes-all (WTA), Rivalry oscillations (RIV), Simultaneous activity (SIM). Blue lines show fixed point branches and the green line shows the maximum of $E_{1} $ & $E_{2} $ on the limit cycle branch. The minimum of RIV branch oscillations is close to zero once away from Hopf bifurcation (not shown). (**B**) Details of the diagram are shown in a zoomed panel. The period of oscillations on the unstable limit cycle branch shown with green dashed lines increase sharply as we move toward a critical parameter value $h\approx 4.22843$ and continuation fails. The dotted green line shows the assumed location of a branch segment that proved difficult to compute due to the orbits having large period. The sequence of bifurcations that transform the system from WTA to RIV periodic solutions has not been described previously. (**C**) Time histories associated with each dynamical behavior: WTA ($h=1$), RIV ($h=4.3$), SIM ($h=15$). Other parameters: $g=1.5$, $J_{\mathrm{HL}}=J_{\mathrm{VR}}=10$

Previously, three main types of dynamical behaviors were found in many models of rivalry with fixed inputs (including the Wilson model): Winner-take-all (WTA), Rivalry oscillations (RIV), Simultaneous activity (SIM). The bifurcation diagram with fixed inhibition strength $g=1.5$, and varying adaptation strength *h*, is presented in Fig. 3(A). A trivial symmetric equilibrium always exists, which is stable for large values of adaptation strength and corresponds to simultaneous activity (SIM) (Fig. 3(C)). This equilibrium loses its stability with decreasing adaptation strength and a stable limit cycle emerges from a supercritical Hopf bifurcation (H). These relaxation oscillations correspond to rivalry (RIV). For *h* increasing from zero on the symmetric unstable branch, a pair of unstable fixed point branches emerge at a pitchfork bifurcation (PF) with the $E_{1}=E_{2}$ symmetry broken. The complementary branches undergo fold bifurcations (L) nearby but remain unstable. These two unstable fixed point branches go through two supercritical Hopf bifurcations and become stable. These two stable equilibria create a bistable parameter range known as winner-take-all (WTA), which exists for small *h*. The qualitative transformation of the system from RIV to WTA remains unclear and must involve as yet undetermined bifurcations at changes in stability on the RIV branch (Fig. 3(B), green curve).

**Figure 4:**
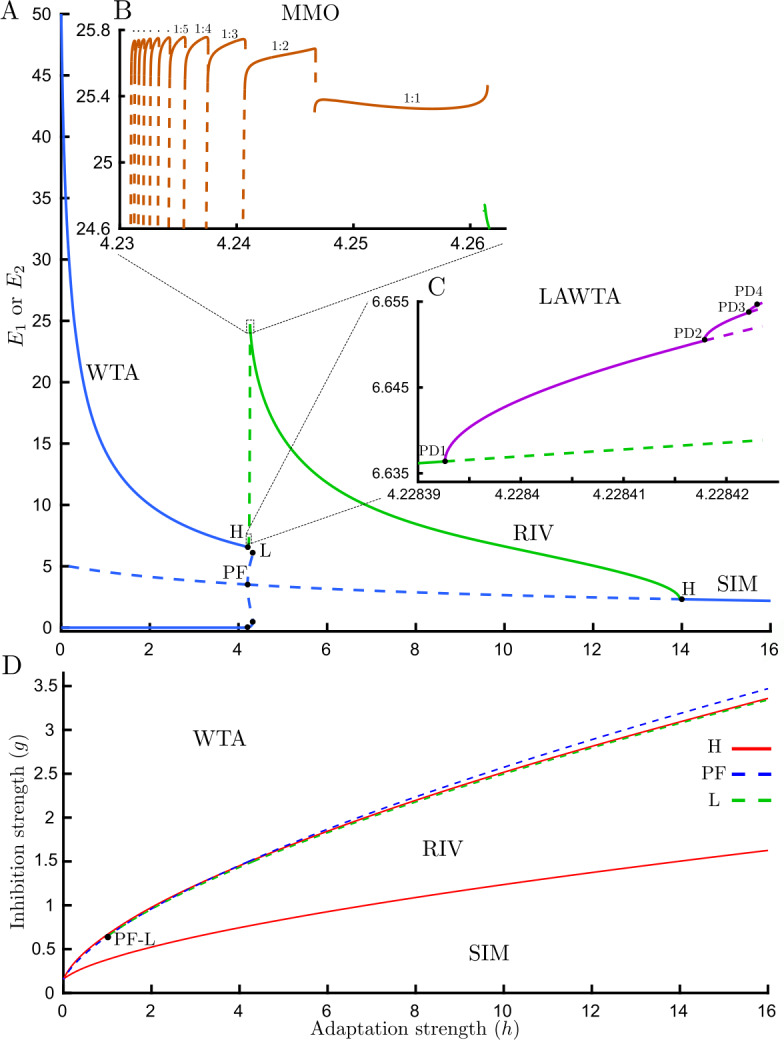
Detailed bifurcation analysis for traditional rivalry. (**A**) Bifurcation diagram of the Wilson model (1) with fixed inputs varying adaptation strength *h*, $g=1.5$. In addition to WTA, RIV, and SIM, two other regions with different dynamical behavior are revealed: (**B**) Mixed-mode oscillations (MMOs) emerging from high amplitude relaxation oscillations (RIV) with discontinuous transitions between segments. Each period of these MMOs has one high and one or more low-amplitude oscillations (see Fig. 6 for time histories). On MMO branches n:m defines the n high to m low-amplitude oscillations ratio. The number of low-amplitude oscillations starts from one and is increased by one as we move down in the bifurcation parameter. (**C**) Low amplitude winner-take-all (LAWTA) oscillations emerge from supercritical Hopf bifurcation on the WTA branch and by further increasing the bifurcation parameter, a cascade of period-doubling bifurcations emerges. Panels B and C show the maximum of $E_{1} $ & $E_{2} $ on the limit cycle branches. The minimum of MMOs is close to zero. (**D**) Boundaries of different dynamical behaviors are shown in parameter space $(h,g)$. The region with the periodic solution (RIV) is confined by Hopf bifurcation (red solid line) from beneath and by fold bifurcation (L, green dashed line) from above. Other parameters: $J_{\mathrm{HL}}=J_{\mathrm{VR}}=10$

**Figure 5:**
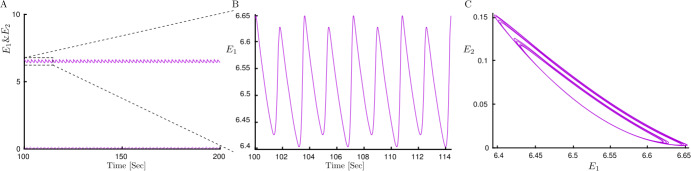
LAWTA oscillations time histories for traditional rivalry. (**A**) Time history of LAWTA oscillatory activity for two neural populations (solid and dashed lines correspondingly). (**B**) One period of LAWTA after PD3 in Fig. 4(C). LAWTA has eight different high and low peaks. (**C**) Limit cycle in the $E_{1}$–$E_{2}$ plane. Parameters: $h=4.22842214$, $g=1.5$, $J_{\mathrm{HL}}=J_{\mathrm{VR}}=10$

In this study, a more complete numerical bifurcation analysis has been carried out to find other stable solutions in the small parameter gap between the WTA and RIV (Fig. 3(B)). As seen in Fig. 4(A), a pair of stable limit cycles emerge from two supercritical Hopf bifurcations (on upper and lower WTA branches) as adaptation strength is increased. These low-amplitude oscillations move around the top-most and bottom-most of the five equilibrium branches existing between PF and L. We call this dynamical behavior low-amplitude-winner-take-all (LAWTA) since there is bistability like WTA behavior; however, the stable states are oscillatory solutions with very small amplitude around asymmetric unstable equilibria (Fig. 4(A)). By further increasing adaptation strength, a cascade of period-doubling bifurcations emerges from the LAWTA branch (Fig. 4(C)). For example, one period of the original limit cycle emerging from a supercritical Hopf bifurcation looks like a sinusoidal signal with one peak and one trough. After the first period-doubling bifurcation (PD), one period of oscillations will have two peaks and two troughs with tiny differences between the peak and trough amplitudes. As seen in Fig. 5, even after three PD bifurcations, the difference in amplitude between the 8 peaks remains small.

**Figure 6:**
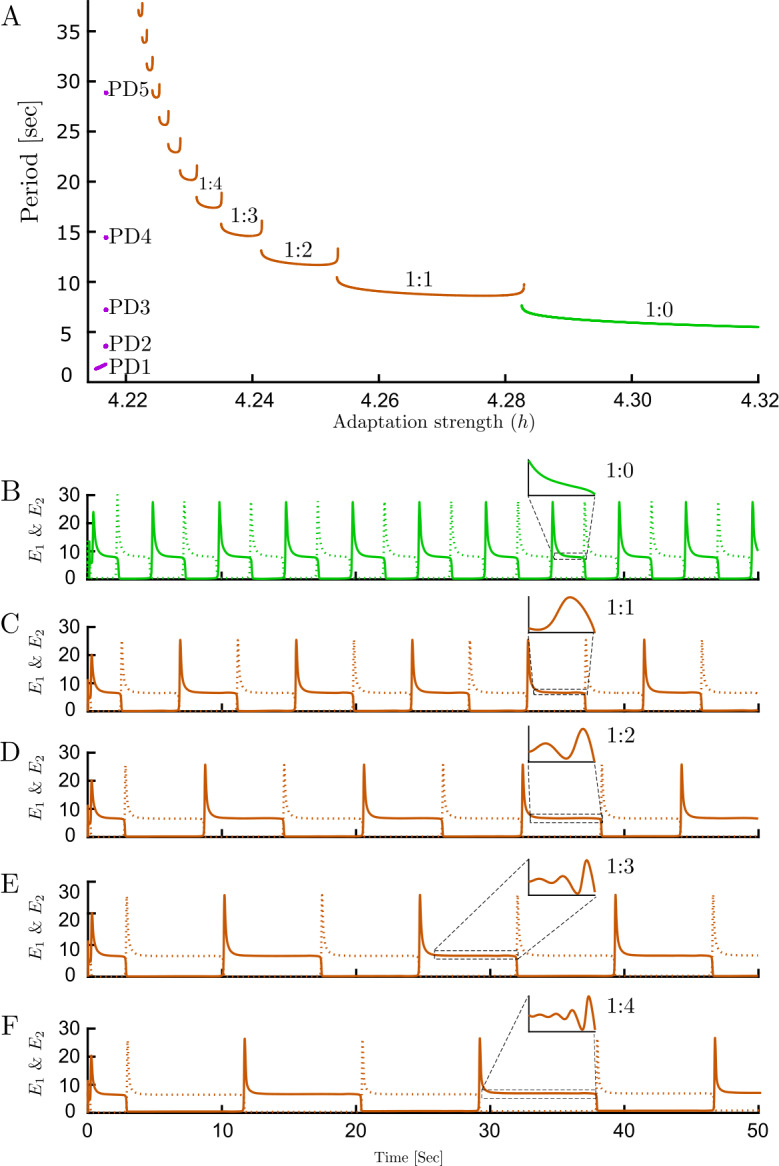
Periods of oscillatory states and MMOs time histories for traditional rivalry. (**A**) Periods of oscillations for three types of oscillatory dynamics: LAWTA (purple), MMOs (brown) and rivalry oscillations (green). The period of oscillations increase sharply as we move toward a critical parameter value $h\approx 4.22843$ from either side. (**B**–**F**) Time histories associated with different branch segments of MMOs with different adaptation strength in each panel. Number of low-amplitude oscillations increases as adaptation strength is decreased: (**B**) RIV ($h=4.3$) (**C**) One ($h=4.26$) (**D**) two ($h=4.243$) (**E**) three ($h=4.24$) (**F**) four ($h=4.237$) low-amplitude oscillations in one period. On MMOs branches n:m defines the n high to m low-amplitude oscillations ratio. Other parameters: $g=1.5$, $J_{\mathrm{HL}}=J_{\mathrm{VR}}=10$

On the other side, the limit cycle emerging from the supercritical Hopf bifurcation (green branch labeled RIV in Fig. 3(A–B)) becomes unstable as adaptation strength decreases; however, the continuation software does not detect any bifurcation where the stability changes. This appears to be a global bifurcation which is not detectable by local analysis. We will refer to this putative global bifurcation at the change in stability at the RIV branch as the global bifurcation. Without any specific bifurcation in hand, we cannot follow any emerging stable branch. To tackle this issue we compute the stable periodic solution (assuming one exists, using numerical integration) for a specific value of adaptation strength (in a range between last PD and the change in stability on the RIV branch) and then follow any periodic solution branch using numerical continuation. On the right side of the global bifurcation, time simulations show relaxation oscillations that reach high amplitude rapidly and before relaxing to baseline (Fig. 6(B)). However, on the left side of the change in stability (close to the global bifurcation), we observe that in addition to high amplitude oscillations, one low amplitude oscillation appears (Fig. 6(C)). By further decreasing adaptation strength, there is always one high amplitude oscillation, but the number of low amplitude oscillations increases (Fig. 6(C–F)). The period of these oscillations increase sharply as we move toward a critical parameter value $h\approx 4.22843$ (Fig. 6(A)). This complex behavior is known as mixed-mode oscillations (MMOs) since it is a mixture of low and high amplitude oscillations. Using the approach described above we could compute stable branches on the left side of global bifurcation (Fig. 4(B)). Interestingly, stable solutions occur through a series of discrete branches. The discontinuous transitions from one branch segment to the next are similar to the spike-adding mechanism from [30]. The bifurcation structure here appears similar to the canard-induced MMOs identified in a spiking neuron model [31]. The sharp increase in amplitude of the limit cycle branch over a short parameter range emerging near the critical value of *h* (Fig. 6 caption) suggests the complex behavior in this region is also associated with canards. Bifurcation analysis of another simple rivalry model with 4 ODEs and different nonlinearity revealed a similar structure for MMOs through the interaction of canards and a singular Hopf point [32]. Whilst, there are some similarities with the MMOs found in the present study, a more rigorous approach would be needed to say whether these MMOs are canard-induced, Hopf-induced, or result from an interaction of both mechanisms. The global bifurcation and the transitions between MMO branches remain to be determined. The behaviors reported here are confined to a narrow region of the $(g,h)$ parameter plane near the left-hand locus of Hopf bifurcation (Fig. 4(D)).

Here, a more complete analysis has revealed MMOs emerge from high amplitude RIV oscillations (Fig. 4(B)) and a cascade of period-doubling bifurcations emerge from LAWTA oscillations (Fig. 4(C)) which have not been reported before in the Wilson model. This analysis describes the mechanism of state transition from WTA to RIV which was not clear before. Whilst MMOs have been reported in another model of rivalry [32, 33], the appearance of a stable PD cascade has revealed richer dynamics in the Wilson model which may also play a part in the mechanisms that lead to appearance and disappearance of limit cycles associated with MMOs. An interesting avenue of investigation will be to understand how the low-amplitude PD cascade (Fig. 4(C)) interacts with periodic forcing. This provides the context to fully understand the periodically forced case.

### Bifurcation analysis of binocular rivalry with periodic forcing

#### Flicker (18 Hz) only

**Figure 7:**
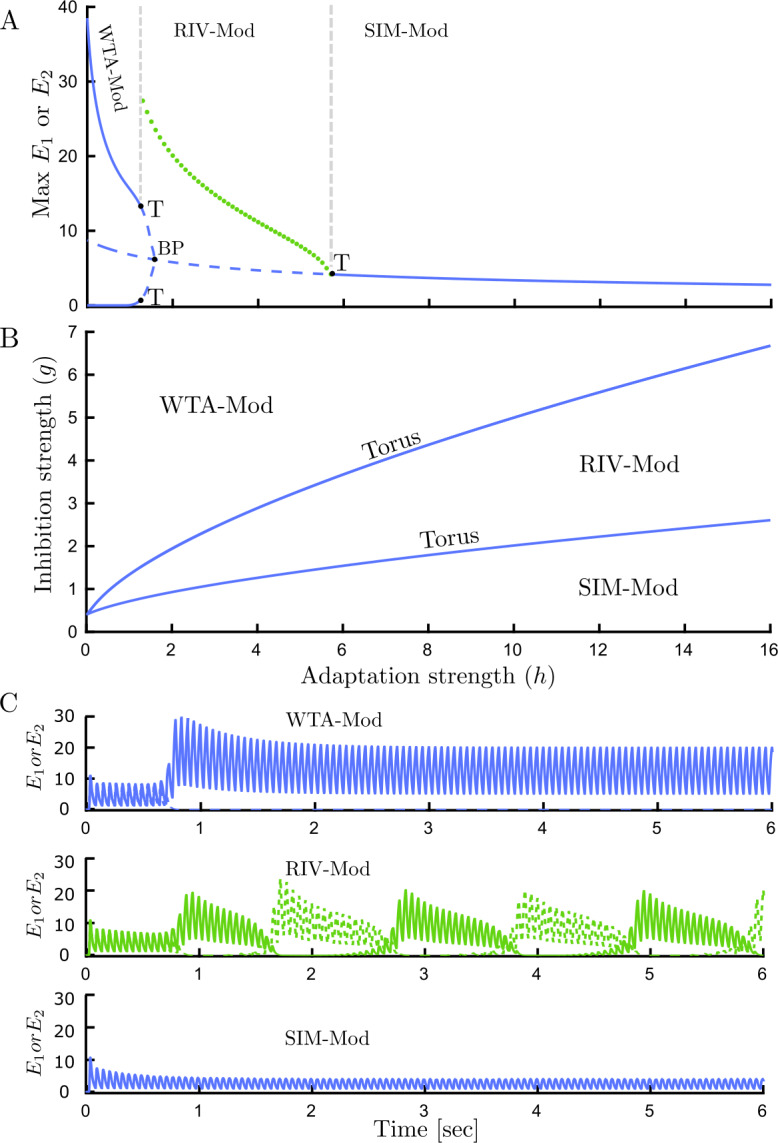
Bifurcation analysis and time histories for the flicker only case. (**A**) Bifurcation diagram for the Wilson model (1) with high frequency periodic forcing (flicker; 18 Hz) varying adaptation strength *h*, $g=1.5$. Three main types of dynamical behaviors are modulated by forcing frequency: (1) Modulated WTA (WTA-Mod), (2) Modulated rivalry (RIV-Mod), (3) Modulated SIM (SIM-Mod). RIV-Mod branch which occurs through supercritical torus bifurcation (T), is associated with slow rivalry alternations. Solid curve: stable limit cycle, dashed curve: unstable limit cycle, filled circles: attracting torus. (**B**) Boundaries of different dynamical behavior with high frequency periodic forcing (flicker 18 Hz) are shown in parameter space $(h,g)$. The region with the RIV-Mod solution is confined by the curve of torus bifurcation. (**C**) Firing activity of each competing population $E_{1}$ (solid lines) and $E_{2}$ (dashed lines) with high frequency periodic forcing; flicker 18 Hz and different adaptation strength *h*: WTA-Mod regime with $h=0.5$, RIV-Mod regime with $h=2$, SIM-Mod regime with $h=6$. Other parameters: $[J_{\mathrm{HL}}]_{\max }=[J_{\mathrm{VR}}]_{\max }=10$

Bifurcation analysis with the flickering stimulus shows that periodic forcing with high frequency (e.g. 18 Hz) modulates the three main types of behaviors that occur with fixed inputs. Instead of WTA and SIM fixed point branches in traditional rivalry with a fixed stimulus (Fig. 3(A)), modulated WTA (WTA-Mod) and modulated SIM (SIM-Mod) periodic solution branches are found with the flickering stimulus (Fig. 7(A)). Subsequently, the SIM-Mod branch undergoes a torus bifurcation (T) giving rise to a torus branch with aperiodic oscillations corresponding to modulated slow rivalry alternations (RIV-Mod). Following the torus bifurcation in the $(g,h)$ parameter plane defines the boundary of rivalry oscillations (Fig. 7(B)). The locus of a pitchfork bifurcation (BP) remains close to the left-hand torus curve (Fig. 7(B), not shown). It appears that the MMO branches and PD cascade identified for fixed inputs disappear with the introduction of flicker. This analysis found evidence that slow rivalry alternations RIV-Mod can exist at parameter values adjacent to regions where stimulus-induced oscillations exist (SIM-Mod). Time histories of these dynamical behaviors are shown in (Fig. 7(C)). These results are consistent with experiments: Flicker stimuli do not differ from the traditional rivalry case [13].

#### Swap (1.5 Hz) only

**Figure 8:**
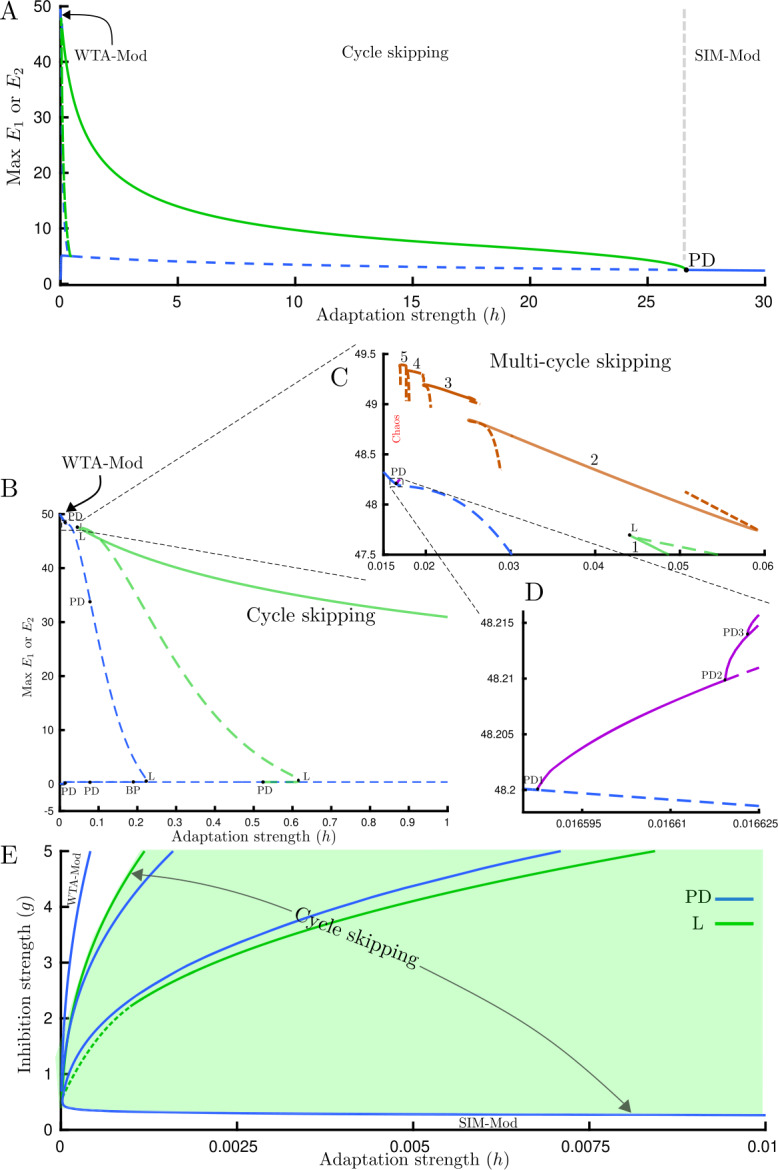
Bifurcation analysis for swap only case. (**A**) Bifurcation diagram for the Wilson model (1) with low frequency periodic forcing (swap; 1.5 Hz) varying adaptation strength *h* at $g=1.5$. Dynamical behavior for large values of adaptation strength is modulated SIM (SIM-Mod). Cycle skipping behavior appears through a period-doubling bifurcation (PD) in which every population only responds to every other stimulus onset in turn. There also exist a pair of stable limit cycles for very small values of adaptation strength which corresponds to modulated WTA (WTA-Mod). Solid curve: stable limit cycle, dashed curve: unstable limit cycle. (**B**) Detailed bifurcation diagram for the Wilson model with low frequency periodic forcing (swap; 1.5 Hz) varying adaptation strength *h* at $g=25$. (**C**) Multi-cycle skipping occurs through discontinuous branches. The number of cycles skipped between switches increases by one as we move left from one branch segment to the next. (**D**) A cascade of period-doubling bifurcations that leads to chaos. In panels C and D, the ordinate shows maximum of $E_{1} $ & $E_{2} $. (**E**) Boundaries of different dynamical behaviors with low frequency periodic forcing (swap 1.5 Hz) are shown in parameter space $(h,g)$. The region with the cycle skipping solution is confined by period-doubling (PD) bifurcations from beneath and by fold bifurcation from above (marked with arrows). Other parameters: $[J_{\mathrm{HL}}]_{\max }=[J_{\mathrm{VR}}]_{\max }=10$

**Figure 9:**
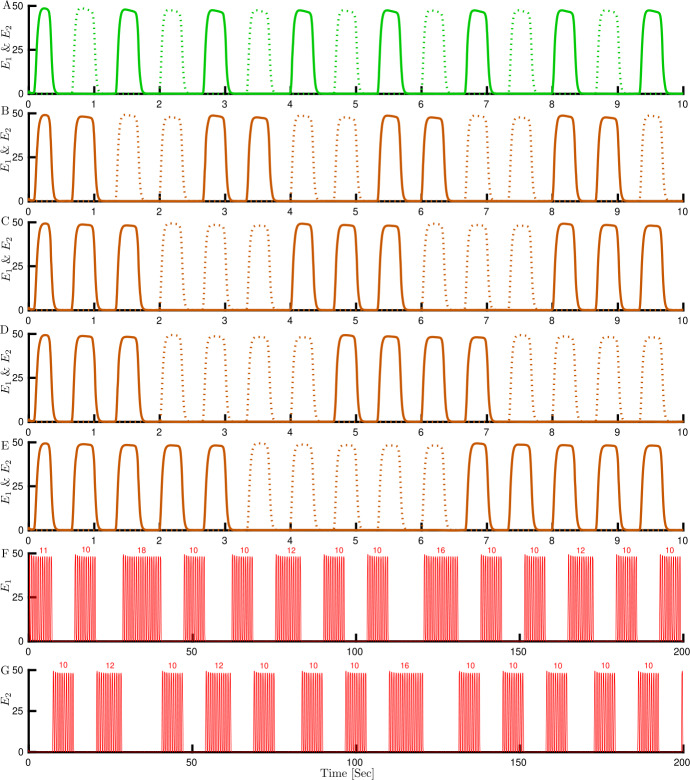
Cycle skipping, multi-cycle skipping and chaos time histories for the Swap only case. Firing activity of each competing population $E_{1}$ (solid lines) and $E_{2}$ (dashed lines) with low frequency periodic forcing; swap 1.5 Hz and different adaptation strength *h*. (**A**) Cycle skipping regime with $h=1$. (**B**–**E**) Multi-cycle skipping regime with variable number of cycles to respond and skip between switches. (**B**) $h=0.03$, (**C**) $h=0.02$, (**D**) $h=0.0185$, (**E**) $h=0.017$. Chaotic firing activity of (**F**) $E_{1}$ and (**G**) $E_{2}$ with irregular number of cycles to respond or skip between switches, $h=0.01663$. Other parameters: $g=25$, $[J_{\mathrm{HL}}]_{\max }=[J_{\mathrm{VR}}]_{\max }=10$

The dynamical behavior with low frequency periodic forcing (around 1.5 Hz, so-called swap) is different, and in addition to WTA-Mod behavior (for small values of adaptation strength) and SIM-Mod (for large values of adaptation strength), cycle skipping occurs through a PD bifurcation on the SIM-Mod branch (Fig. 8(A)). Cycle skipping refers to the response of each competing population to every other stimulus onset. This means two populations respond in turn to stimulus cycles and while one has high activity during a cycle the other one stays inactive (Fig. 9(A)). We note that the period of the period-doubled solution is 1.333 s and is plausibly the result of a resonance with the fixed-input limit-cycle, which has a period >2 s, for larger values of *h* [11]. SIM-Mod and cycle skipping have been reported in a simpler model of rivalry [34] and via direct simulations of the Wilson model in [25].

As seen in (Fig. 8(B–C)), the cycle skipping branch loses stability at a fold bifurcation (L) for decreasing *h*. For *h* increasing, branching off from the PD point on the WTA-mod branch leads to a cascade of PD bifurcations (Fig. 8(D)). In order to find the possible stable periodic branches between the last PD and the fold bifurcation, we compute the stable periodic solution (assuming it exists) using numerical integration for a specific value of adaptation strength, and then start continuation from this solution. With this approach, we found a family of discontinuous branches, which correspond to multi-cycle skipping (Fig. 8(C)). On these branches, the number of stimulus cycles between switches of activity from one population to the other is variable and increases by one as the bifurcation parameter decreases (Fig. 9(A–E)). Here we found a small region of bistability between the one-cycle skipping and two-cycle skipping behaviors (at around $h=0.05 $ in Fig. 8(C)). Therefore, in the full hierarchical model it would be possible to find an asymmetric solution where the HL-VR units at the first stage behave differently. However, as explained in the discussion the model normally operates close to the SIM-Mod region, which is far away in parameter space from the cycle skipping region with bistability.

Another interesting behavior is the appearance of a chaotic attractor in a parameter range between the PD cascade and multi-cycle skipping family branches (Fig. 8(C)). Figure 9(F–G) represents chaotic firing activity for each population in a 200 s simulation. The number of cycles between switches does not show any regular or repeating pattern.

#### Flicker (18 Hz) & (1.5 Hz) Swap

**Figure 10:**
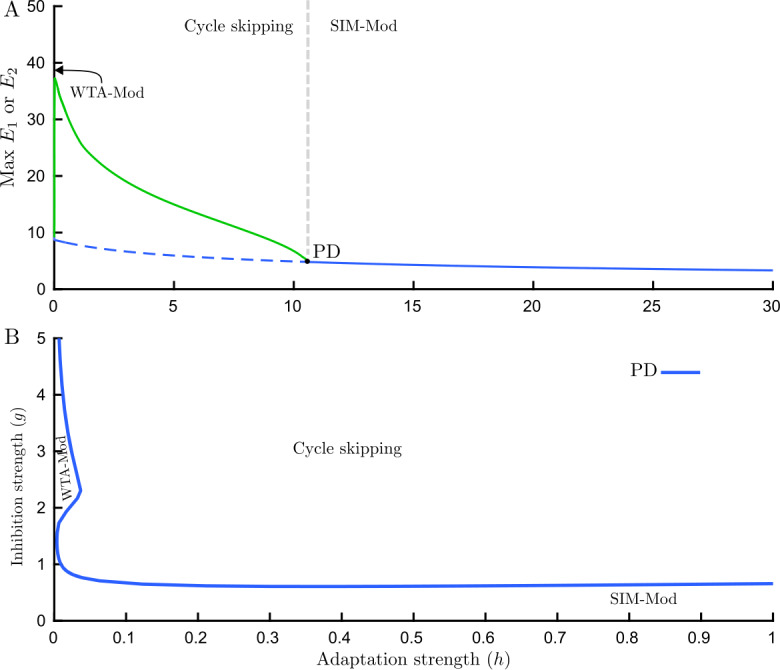
Bifurcation analysis for F&S rivalry. (**A**) Bifurcation diagram for the Wilson model (1) with high frequency flickering (18 Hz) and low frequency swap (1.5 Hz) varying adaptation strength *h* at $g=1.5$. Dynamical behavior for large values of adaptation strength is modulated SIM (SIM-Mod). Cycle skipping behavior appears through period-doubling bifurcation (PD) in which every population only responds to every other stimulus onset in turn. There also exist a pair of stable limit cycles for very small values of adaptation strength which corresponds to modulated WTA (WTA-Mod), not visible at this scale. Solid curve: stable limit cycle, dashed curve: unstable limit cycle. (**B**) Boundaries of different dynamical behaviors are shown in parameter space $(h,g)$. The region with cycle skipping solution is confined by period-doubling (PD) bifurcations. Other parameters: $[J_{\mathrm{HL}}]_{\max }=[J_{\mathrm{VR}}]_{\max }=10$

**Figure 11:**
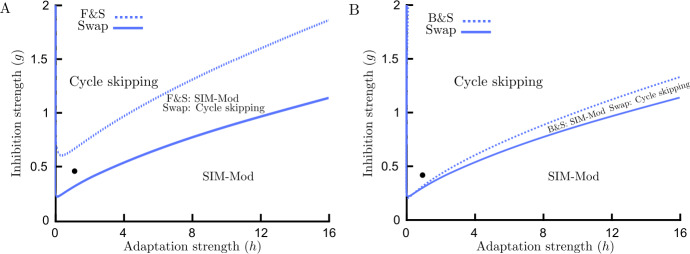
Why does the Wilson model produce slow alternations with the F&S, but not with swap only and B&S stimuli? Two parameter bifurcation diagram for the Wilson model (1) which defines regions with different dynamical behaviors. Blue curves show the curve of PD bifurcation and the boundary for cycle skipping behavior. Below this curve the dynamical behavior is SIM-Mod. The black dot defines the point at which the Wilson model operates. (**A**) Comparison of swap and F&S cases. As seen, for swap only stimuli the monocular layer operates in the cycle skipping regime; however, for F&S stimuli it operates in the SIM-Mod regime. The binocular layer for F&S stimuli is effectively stimulated with flickering stimuli and by selecting the current inhibition strength it is possible to get slow rivalry alternations in the second layer. (**B**) Comparison of swap and B&S cases. Inserting blanks (with 150 ms durations) before swap times, like adding flicker, moves the boundary between Cycle skipping and SIM-Mod regions up in the parameter plane (but to a lesser extent) and the likelihood of being in the SIM-Mod region increases

The bifurcation structure with both high frequency flicker and low frequency swap appears to be analogous to bifurcation structure with swap only case (Fig. 10(A–B)). However, the right-hand PD bifurcation point in the transition from cycle skipping to SIM-Mod moves down in adaptation strength. This is shown in a direct comparison of two-parameter bifurcation diagrams for the F&S and swap only cases in (Fig. 11(A)). In fact, with the same values of adaptation strength that we might expect cycle skipping from swap only case, with F&S stimulus we can get SIM-mod, which turns out to be critical for obtaining slow alternations, see discussion.

#### Blanks (150 ms) & (1.5 Hz) Swap

Our result shows that the effect of blank insertion before swap times, similar to the effect of adding flicker to swap stimuli, moves the PD bifurcation point down the bifurcation parameter *h* (Fig. 11(B)), but to a much lesser extent than the introduction of flicker; compare Fig. 11(A) and (B).

## Discussion

### Summary

Earlier work with models of bistable perception has identified parameter ranges with winner-take-all dynamics (WTA), rivalry oscillations (RIV) and simultaneous activity (SIM) [35, 36]. Such regimes are known for the Wilson model as input strength is increased [11]. Our results show that the Wilson model with fixed inputs is capable of generating complex dynamical behaviors such as MMOs and LAWTA oscillations, previously not reported. We have also built a framework for studying rivalry models with periodic inputs using numerical continuation. Given symmetry between orientations (0° & 90°) and eyes (L&R), and the feed-forward structure of the hierarchical Wilson model, it is sufficient to study one pair of units in the monocular layer. We found that periodic forcing with high frequency (e.g. 18 Hz, known as flicker) modulates the three main types of behaviors that occur with fixed inputs with forcing frequency (WTA-Mod, RIV-Mod, SIM-Mod). However, the dynamical behavior changes with low frequency periodic forcing (around 1.5 Hz, so-called swap), and in addition to WTA-Mod and SIM-Mod, cycle skipping and multi-cycle skipping behavior exist which can lead to chaotic dynamics. Cycle skipping behavior with swap stimuli (1.5 Hz fast alternations) is not consistent with experiments [13, 37], where we expect slow rivalry alternation (duration around 2 s). In order to understand the dynamics of the full hierarchical model with periodic forcing, we should consider that the inputs for the second layer of the Wilson model are the responses of populations selective to the same orientation from the first monocular layer (Fig. 1(A)). For example, if the isolated subunits in the first layer are in the SIM-Mod regime (Fig. 11(A)), the stimuli for the second layer will look like a fixed stimulus to the second layer (where SIM-Mod occurs in the first layer for the Swap only stimulus) or a flickering stimulus (where SIM-Mod occurs in the first layer for the F&S stimulus). This means the second layer is effectively stimulated with traditional or flickering stimuli and by selecting the current inhibition strength it is possible to get slow rivalry alternations in the second layer. This provides a deeper explanation of how the Wilson model produces slow alternations with the F&S (but not Swap only stimulus).

### Rivalry model complexity and comparison with other models

There are other computational models that can capture properties of perceptual dominance durations in both types of experiments (traditional experiments and F&S experiments) [24, 25]. Brascamp’s model, with less complexity than Wilson’s, has only one layer of monocular units with the extension of inhibition to within-eye and cross-eye iso-orientation connections [38, 39]. The model proposed by Brascamp et al. correctly reflects the fluctuations in monocular neurons; however, their model generates slow alternations only when the blank intervals before swaps are short. The Li et al. model [25] with another layer of attentional modulation (in addition to monocular and binocular layers) is the most sophisticated binocular rivalry model, accounting for a wide range of phenomena but with greatly increased complexity (14 ODEs).

**Figure 12:**
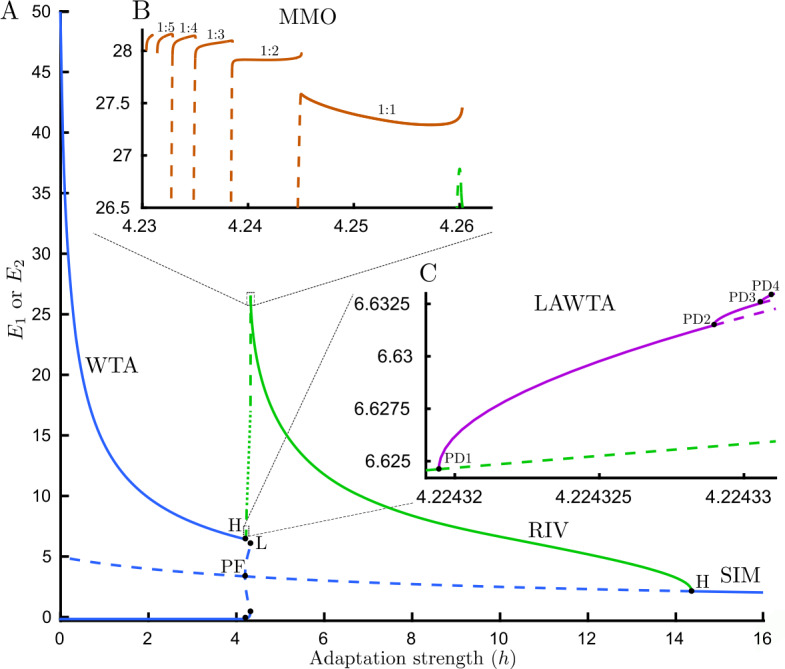
(**A**) Detailed bifurcation diagram of the reduced Wilson model (instantaneous inhibition dynamics, $\tau _{I} \to 0$ in Eq. (1)) with fixed inputs varying adaptation strength *h* at $g=1.5$. The dotted green line shows the assumed location of a branch segment that proved difficult to compute due to the orbits having large period. All complex dynamical behaviors still persist: (**B**) Mixed-mode oscillations (MMOs) with discontinuous transitions between segments. On MMO branches n:m defines the n high to m low-amplitude oscillations ratio. The number of low-amplitude oscillations starts from one and is increased by one as we move down the bifurcation parameter. (**C**) Low amplitude winner-take-all (LAWTA) oscillations emerge from Hopf bifurcation on the WTA branch and by further increasing the bifurcation parameter, a cascade of period-doubling bifurcations emerges. Panels B and C show the maximum of $E_{1} $ & $E_{2} $ on the limit cycle branches

MMOs have already been reported in a simpler rivalry model with four ODEs (without an inhibition equation in each subunit) through interactions of singular Hopf and canard [32, 33]. However, the LAWTA regime emerging through a PD cascade has not been reported before. The question arises as to whether there is a distinct mechanism (from say [32, 33]) specific in the Wilson model that leads to similar MMO dynamics. We found that by assuming instantaneous inhibition dynamics ($\tau _{I} \to 0$ in Eqs. (1)) in the Wilson model, all of the complex behaviors with traditional stimuli still persist (Fig. 12). Thus any differences to the bifurcation structure across different models are likely due to the differences in the nonlinearity processing inputs to each unit. In the Wilson model the Hopf bifurcation is supercritical (subcritical in [32]) and stable branches emerging in a PD cascade appear to terminate (lose stability) at the *h*-value where MMOs first emerge. The large increase in period and the large excursion in phase space required to jump from the PD branch to the full-amplitude MMO branches at a critical value of *h* are consistent with a canard mechanism, but more exotic than reported previously. Indeed, further analysis would be needed to resolve how the PD cascade terminates and the MMO branches emerge through a common mechanism.

### Limitations of the Wilson model

As discussed in [24, 25], the Wilson model is only capable of generating slow perceptual alternations with the F&S stimulus, and it fails to do so with blank intervals inserted before each swap, so-called Blank & Swap (B&S) [26, 27]. Our results show that blank insertion similar to a flickering stimulus moves the boundaries up in the adaptation–inhibition strength plane (Fig. 11(B)), but to a lesser change than F&S. In order to be in the SIM-Mod area, which as discussed before is necessary for slow rivalry alternations at the second stage, adaptation strength needs to be increased or inhibition strength decreased. On the other hand, the existence of cycle skipping behavior with swap stimuli leads to perceptual alternation with a frequency half of the swap rate at the second stage [25], which to our knowledge is not consistent with empirical results [13, 37]. This reported limitation of the Wilson model could simply be avoided if the parameters had been set in a way that the model operated in the SIM-Mod regime. Our result casts a new light on binocular rivalry with periodic forcing and it is now clear that these two limitations of the Wilson model could be fixed by tuning model parameters.

### Implications for experiments

Even though we showed that model parameters can be tuned for consistent behavior with experimental results, our results also predict apparently spurious dynamical behaviors such as cycle skipping with swap stimuli at the monocular level that has not been reported by experimental work. It is possible that this behavior (a 1.5 Hz fast alternation) was not distinguishable from 3 Hz fast alternation in perceptual reports. Future empirical studies could investigate the existence of such behavior at the early stages of visual processing with monocular contributions and at later stages, i.e. higher cortical layers where the activity would propagate.

### Future work

Levelt’s propositions [17] with traditional rivalry are well known. However, they have not been investigated with F&S stimuli in any of the existing models of rivalry. It has been shown in experiments [1] that Levelt’s proposition II holds with F&S stimuli (whilst to our knowledge Levelt’s proposition IV has yet to be investigated). By implementation of these forcing cases with numerical continuation we now have the tools to determine constraints on models such that they are consistent Levelt’s propositions with periodic forcing [2].

The PD cascade found in the traditional rivalry case suggests the existence of chaotic dynamics in a small range of parameter between PD cascade and MMO branches. This could be checked by computing Lyapunov exponents for a continuous-time dynamical system. Note that these low-amplitude oscillations (LAWTA) appear to interact with periodic forcing to produce chaotic dynamics at high amplitude (Fig. 9).

Taking the unforced system and adding periodic inputs we might expect the fine structure from the traditional rivalry case to be washed out. This is true in e.g. the flicker case. However, we find in the swap case that the periodic forcing does interact with the deterministic dynamics to produce a family for cycle skipping branches and chaotic dynamics. Noise plays an important role in rivalry, and this is often considered in models [35]. It remains outside the scope of the current work to explore whether the regions of exotic dynamics found in the traditional case and in the swap case would be washed out by the inclusion of noise. Nevertheless, the introduction of noise to timescale-separated dynamical systems (like the Wilson model) can introduce dynamics not present in the deterministic case, especially local to bifurcations [40]. Interactions between noise and the dynamics may modify the exotic dynamics reported here in an interesting way, and could expand the parameter regions where these states persist.

Our approach for studying perceptual bistability with periodic stimuli is applicable for a range of other stimuli including auditory streaming [41], motion illusions [28] and haptic bistability [42].

### Conclusion

The main conclusions of this work are drawn together and presented here. First, the results of our study show that the transition from slow rivalry alternation to the WTA regime is much more complicated than previously reported. As shown in Shpiro et al. [35], the stochastic characteristic of the dominance durations in the presence of noise are best described near this boundary (transition from RIV to WTA). It would be of interest to see how these complex dynamics (MMOs & LAWTA) will interact with noise. Further analysis is needed to check whether these dynamical regimes persist or are modified in the presence of noise.

Second, several competition models have been proposed to describe binocular rivalry with periodic stimuli, however, interpretation of results from these models are based on a specific set of parameters. Here we introduced a method to assess whether the existing models of binocular rivalry are valid or not in a specific parameter regime and, more generally, to find the parameter regions where these models work. This research provides a framework for either assessing binocular rivalry models for consistency with empirical results, or for better understanding neural dynamics and the mechanisms necessary to implement a minimal binocular rivalry model.

## Abbreviations

BP: Branch Point
B&S: Blanks and Swaps
EEG: Electroencephalogram
fMRI: Functional Magnetic Resonance Imaging
F&S: Flicker and Swaps
H: Hopf Bifurcation
HL: Horizontal Left
HR: Horizontal Right
L: Fold Bifurcation
LAWTA: Low Amplitude Winner-take-all
MMOs: Mixed-mode Oscillations
Mod: Modulated
PD: Period-doubling
PF: Pitchfork Bifurcation
RIV: Rivalry Oscillations
SIM: Simultaneous Activity
T: Torus Bifurcation
VL: Vertical Left
VR: Vertical Right
WTA: Winner-take-all

## References

[1] Logothetis NK, Leopold DA, Sheinberg DL (1996). What is rivalling during binocular rivalry?. Nature.

[2] Wilson HR (2003). Computational evidence for a rivalry hierarchy in vision. Proc Natl Acad Sci.

[3] Blake R, Logothetis NK (2002). Visual competition. Nat Rev Neurosci.

[4] Wilke M, Logothetis NK, Leopold DA (2003). Generalized flash suppression of salient visual targets. Neuron.

[5] Logothetis NK, Schall JD (1989). Neuronal correlates of subjective visual perception. Science.

[6] Leopold DA, Logothetis NK (1996). Activity changes in early visual cortex reflect monkeys’ percepts during binocular rivalry. Nature.

[7] Polonsky A, Blake R, Braun J, Heeger DJ (2000). Neuronal activity in human primary visual cortex correlates with perception during binocular rivalry. Nat Neurosci.

[8] Tong F, Engel SA (2001). Interocular rivalry revealed in the human cortical blind-spot representation. Nature.

[9] Zhang P, Jamison K, Engel S, He B, He S (2011). Binocular rivalry requires visual attention. Neuron.

[10] Laing CR, Chow CC (2002). A spiking neuron model for binocular rivalry. J Comput Neurosci.

[11] Shpiro A, Curtu R, Rinzel J, Rubin N (2007). Dynamical characteristics common to neuronal competition models. J Neurophysiol.

[12] Curtu R, Shpiro A, Rubin N, Rinzel J (2008). Mechanisms for frequency control in neuronal competition models. SIAM J Appl Dyn Syst.

[13] Lee S-H, Blake R (1999). Rival ideas about binocular rivalry. Vis Res.

[14] Sengpiel F, Blakemore C, Harrad R (1995). Interocular suppression in the primary visual cortex: a possible neural basis of binocular rivalry. Vis Res.

[15] Sengpiel F, Freeman T, Blakemore C (1995). Interocular suppression in cat striate cortex is not orientation selective. NeuroReport.

[16] Li B, Peterson MR, Thompson JK, Duong T, Freeman RD (2005). Cross-orientation suppression: monoptic and dichoptic mechanisms are different. J Neurophysiol.

[17] Levelt WJM. On binocular rivalry. Soesterberg, The Netherlands: Institutefor Perception RVO-TNO; 1965.

[18] Cao R, Braun J, Mattia M (2014). Stochastic accumulation by cortical columns may explain the scalar property of multistable perception. Phys Rev Lett.

[19] Lehky SR (1988). An astable multivibrator model of binocular rivalry. Perception.

[20] Blake R (1989). A neural theory of binocular rivalry. Psychol Rev.

[21] Wilson HR (2007). Minimal physiological conditions for binocular rivalry and rivalry memory. Vis Res.

[22] Salinas E (2003). Background synaptic activity as a switch between dynamical states in a network. Neural Comput.

[23] Freeman AW (2005). Multistage model for binocular rivalry. J Neurophysiol.

[24] Brascamp J, Sohn H, Lee S-H, Blake R (2013). A monocular contribution to stimulus rivalry. Proc Natl Acad Sci.

[25] Li H-H, Rankin J, Rinzel J, Carrasco M, Heeger DJ (2017). Attention model of binocular rivalry. Proc Natl Acad Sci.

[26] van Boxtel JJ, Knapen T, Erkelens CJ, van Ee R (2008). Removal of monocular interactions equates rivalry behavior for monocular, binocular, and stimulus rivalries. J Vis.

[27] Denison RN, Silver MA (2012). Distinct contributions of the magnocellular and parvocellular visual streams to perceptual selection. J Cogn Neurosci.

[28] Vattikuti S, Thangaraj P, Xie HW, Gotts SJ, Martin A, Chow CC (2016). Canonical cortical circuit model explains rivalry, intermittent rivalry, and rivalry memory. PLoS Comput Biol.

[29] Doedel EJ, Fairgrieve TF, Sandstede B, Champneys AR, Kuznetsov YA, Wang X. Auto-07p: continuation and bifurcation software for ordinary differential equations (software package). 2007.

[30] Nowacki J, Osinga HM, Tsaneva-Atanasova K (2012). Dynamical systems analysis of spike-adding mechanisms in transient bursts. J Math Neurosci.

[31] Ermentrout B, Wechselberger M (2009). Canards, clusters, and synchronization in a weakly coupled interneuron model. SIAM J Appl Dyn Syst.

[32] Curtu R, Rubin J (2011). Interaction of canard and singular Hopf mechanisms in a neural model. SIAM J Appl Dyn Syst.

[33] Curtu R (2010). Singular Hopf bifurcations and mixed-mode oscillations in a two-cell inhibitory neural network. Phys D: Nonlinear Phenom.

[34] Jayasuriya S, Kilpatrick ZP (2012). Effects of time-dependent stimuli in a competitive neural network model of perceptual rivalry. Bull Math Biol.

[35] Shpiro A, Moreno-Bote R, Rubin N, Rinzel J (2009). Balance between noise and adaptation in competition models of perceptual bistability. J Comput Neurosci.

[36] Seely J, Chow CC (2011). Role of mutual inhibition in binocular rivalry. J Neurophysiol.

[37] Blake R, Westendorf DH, Overton R (1980). What is suppressed during binocular rivalry?. Perception.

[38] Baker DH, Meese TS, Summers RJ (2007). Psychophysical evidence for two routes to suppression before binocular summation of signals in human vision. Neuroscience.

[39] Moradi F, Heeger DJ (2009). Inter-ocular contrast normalization in human visual cortex. J Vis.

[40] Berglund N, Gentz B (2008). Stochastic dynamic bifurcations and excitability. Stochastic methods in neuroscience.

[41] Rankin J, Sussman E, Rinzel J (2015). Neuromechanistic model of auditory bistability. PLoS Comput Biol.

[42] Carter O, Konkle T, Wang Q, Hayward V, Moore C (2008). Tactile rivalry demonstrated with an ambiguous apparent-motion quartet. Curr Biol.

